# Clinicodemographic Profile of Children with Seizures in a Tertiary Care Hospital: A Cross-Sectional Observational Study

**DOI:** 10.1155/2017/1524548

**Published:** 2017-06-21

**Authors:** Nagendra Chaudhary, Murli Manohar Gupta, Sandeep Shrestha, Santosh Pathak, Om Prakash Kurmi, B. D. Bhatia, K. N. Agarwal

**Affiliations:** ^1^Department of Pediatrics, Universal College of Medical Sciences, Bhairahawa 32900, Nepal; ^2^Chitwan Medical College, Bharatpur 44200, Nepal; ^3^Centre for Population Health and Research (CPR), Bhairahawa 32900, Nepal; ^4^Nuffield Department of Population Health, Clinical Trial Service Unit & Epidemiological Studies Unit, University of Oxford, Oxford, UK

## Abstract

Seizures are one of the common causes for hospital admissions in children with significant mortality and morbidity. This study was conducted to study the prevalence and clinicodemographic profile of children with seizures in a tertiary care hospital of western Nepal. This prospective cross-sectional study conducted over a period of 2 years included all admitted children (2 months–16 years) with seizures. Among 4962 admitted children, seizures were present in 3.4% (*n* = 168) of children, with male preponderance. 138 (82.1%) children had generalized tonic-clonic seizures (GTCS) and 30 (17.9%) children had partial seizures. GTCS were more common than partial seizures in both sexes (male = 82.7%; female = 81.2%) and age groups. There was no statistical significance in the distribution of seizures (GTCS and partial seizures) with sexes (*P* = 0.813) and age groups (*P* = 0.955). Mean ages of children having GTCS and partial seizures were 8.2 ± 4.6 years and 8.2 ± 4.2 years, respectively. Loss of consciousness (55.4%), fever (39.9%), vomiting (35.1%), and headache (16.1%) were common complaints in seizure patients. Significant number of GTCS cases had fever (*P* = 0.041) and neurocysticercosis (*n* = 72; 43%) was the most common etiology in seizure patients. Idiopathic epilepsy (38 (22.6%)), meningoencephalitis (26 (15.5%)), and febrile convulsions (14 (8.33%)) were other leading disorders in children with seizures.

## 1. Introduction

Seizure, a transient occurrence of signs and/or symptoms resulting from abnormal excessive or synchronous neuronal activity in the brain, is an important cause for hospital admissions in children from developing countries with increased prevalence in younger children [1, 2]. Studies suggest that around 4–10% of children have an experience of seizure before 16 years of age, where 1/5th of total children with unprovoked seizures may develop epilepsy [3]. Epilepsy in Nepal remains a huge challenge with prevalence of seven per 1000 population [4].

Neonatal seizures (infections, birth asphyxia, and metabolic causes), febrile convulsions, meningitis, viral encephalitis, neurocysticercosis, cerebral malaria, and epilepsy (symptomatic, cryptogenic, and idiopathic) are common causes of acute seizures in children [5–10]. Febrile seizures occurring commonly between 6 months and 5 years of age account for 2–5% of all children experiencing first episode of seizure before 5 years of age. Infections alone can be the major cause of seizures in developing nations [3, 11].

Neuroimaging (CT scan/MRI) plays an important role in the etiological diagnosis of seizures. Generally, neuroimaging is not necessary in well-appearing children after a first, unprovoked nonfebrile seizure. It has an important role in children with focal seizure or persistent seizure activity, focal neurologic deficit, neurocutaneous disorder, signs of elevated intracranial pressure, VP shunting, trauma, or travelling to cysticercosis endemic countries [12–14].

Proper diagnosis, classification, and management are always challenging in a child with seizure. The problem is even more complicated in resource-limited countries like Nepal due to lack of proper investigations and technologies in many hospitals. There are only limited studies based on clinicodemographic profile, types, and etiological causes of seizures in children in western Nepal. This cross-sectional observational study was therefore conducted to study the sociodemographic profile, clinical characteristics, neuroimaging, and use of antiepileptic drugs in children presenting with seizures.

## 2. Materials and Methods

This was a prospective cross-sectional study conducted at Universal College of Medical Sciences, Bhairahawa, Nepal, a tertiary level hospital situated in western Nepal. All admitted cases (0–16 years) to pediatrics ward/PICU presenting with seizures, both unprovoked and symptomatic (acute and remote), were enrolled over a period of 2 years from 1 August 2014 to 31 July 2016. The study was approved by the institute's ethical review board (IRB). The objective of the study was to determine the prevalence of seizures in pediatric hospital admissions and to study the clinicodemographic profile of those children. Neonatal seizures admitted in neonatal intensive care unit were not included for the analysis. Data was recorded in predesigned proforma and analyzed using STATA v13. Association between participants' characteristics and seizures was carried out using multivariate logistic regression. Chi-square test was used for categorical samples, whereas Student's *t*-test was used for noncategorical samples. *P* value of less than 0.05 was considered statistically significant.

## 3. Results

**Figure 1:**
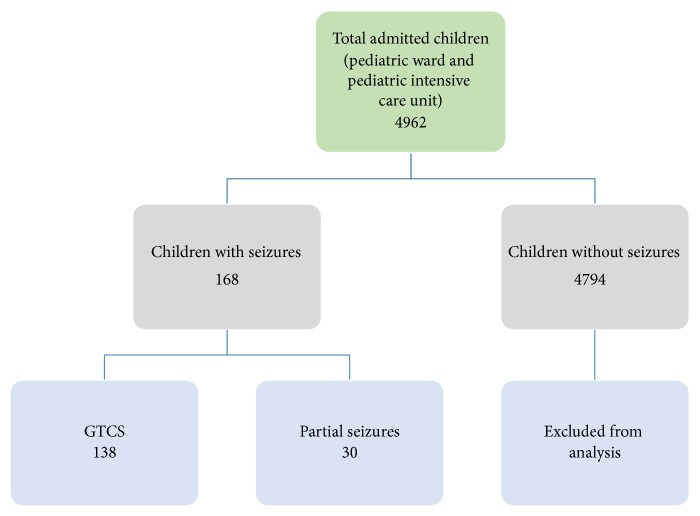
Work plan of admitted children.

Among 4962 children admitted to the paediatric department over a period of two years, 168 children fulfilled the inclusion criteria and were subjected to analysis (Figure 1). Seizures were present in 168 children (104 boys and 64 girls) with youngest age of 2 months. 138 (82.1%) children had generalized tonic-clonic seizures and 30 (17.9%) children had partial seizures. GTCS were more common than partial seizures in both sexes (M = 82.7%; F = 81.2%). There was no statistical significance in the distribution of seizures (GTCS and partial) with sexes (*P* = 0.813). The mean ages of children having GTCS and partial seizures were 8.2 ± 4.6 years and 8.2 ± 4.2 years, respectively (Table 1).

Children having seizures were subdivided into 4 age groups: 0–4 years, 5–8 years, 9–12 years, and 13–16 years. GTCS were again more common in all the age groups when compared to partial seizures. There was no statistical significance between the different age groups and seizure semiology (*P* = 0.955).

The mean values of different biochemical parameters in children having GTCS and partial seizures are depicted in Table 2.

Loss of consciousness (55.4%), fever (39.9%), vomiting (35.1), and headache (16.1%) were four leading clinical complaints in admitted seizure patients, whereas speech disorder (2.4%) was the least common complaint. Among 67 children having fever, 60 (89%) had GTCS, while 7 (10.5%) had partial seizures, which was statistically significant (*P* = 0.041), suggesting that febrile children presented more commonly with GTCS. There was no statistical significance in occurrence of other clinical features (vomiting, headache, meningeal irritation, unconsciousness, and speech disorder) in children with GTCS and partial seizures (Table 3).

Neuroimaging was done in all 168 seizure patients, where 73 (43.4%) were normal and 72 (42.9%) showed neurocysticercosis. The children with normal neuroimaging findings had cerebral palsy (3), febrile convulsion (14), idiopathic epilepsy (38), and meningoencephalitis (18). Remaining 23 abnormal CT head findings were meningoencephalitis (8), acute disseminated encephalomyelitis (3), traumatic head injury (1), gliotic lesion (2), intracranial space occupying lesion (tumor) (2), and structural brain abnormalities (7) (Table 4).  Table 5 shows antiepileptic drugs used in children with GTCS and partial seizures.

## 4. Discussion

Seizures are more common in younger children compared to older ones with male preponderance [15].

In our study, we too observed the higher prevalence of seizures in younger age groups (0–4 years > 13–16 years) with male predominance (male/female = 1.6 : 1). The prevalence of seizures was 3.4% (168 out of 4962 admissions) in our study. A recent study conducted in western region of the country showed the prevalence of seizures to be higher than that in our study (12.7%) with male-to-female ratio almost similar to our study [16]. A study form South India also suggested males being predominant in children with seizures [17]. Another study conducted in the hilly part of Nepal (Kathmandu valley) showed the prevalence of seizures to be 10.2%. The prevalence of seizures may even increase in younger age groups if neonatal seizures (due to sepsis, birth asphyxia, hypoxic ischemic encephalopathy, and metabolic causes) are included. A study conducted in Kenya showed the prevalence of seizures to be almost 18.3%. The reason for higher occurrence of seizures in children as compared to our study may be due to inclusion of newborns having seizures in the Kenya study [6]. In our study, children below 2 months of age were not included.

138 (82%) of all children with seizures had generalized tonic-clonic seizures, whereas the remaining 30 (17.9%) had partial seizures. Other semiologies (absence and atonic seizures) were not seen in the present study. An attempt was made by Shakya et al. to study the relative frequencies of various epileptic seizures and the age at onset of different seizure types in Nepalese children in 2001-2002 on 50 children diagnosed as epilepsy [18]. Generalized seizures (78%) were 3.54 times commoner than partial seizures (22%), which was almost similar to our study. They reported generalized tonic-clonic seizure (36%) as the most common seizure type followed by tonic type (16%), complex partial type (14%), atonic type (12%), and absence (10%), respectively. They also found that the peak age of onset for partial seizures was less than 6 years, while primary generalized seizure was more frequently seen in age group of 2–10 years. Study conducted by Saravanan showed that around two-thirds of children with seizure symptomatology were below 6 years of age [17]. In our study, the mean age of onset for both seizure types was about 8 years, suggesting that this age has maximum number of children being admitted to hospital with seizures. Study done by Adhikari et al. also suggested that majority (69.9%) of children with seizures had generalized tonic-clonic type followed by partial seizures (19.8%). Many studies conducted previously also suggest the high prevalence of GTCS in comparison to partial seizures [6, 9, 17, 19].

Fever was present in 67 (39.9%) children with majority (60 (89.5%)) having GTCS (*P* = 0.041). Ojha and Aryal reported fever with seizures in 75.5% of cases with febrile seizures as the most common etiology [20].

Fever with seizure frequency was 53.5% in a similar study conducted in western region of the country by Adhikari et al. A South Indian study reported the presence of fever in 51.5% of children. In the present study, unconsciousness (93 (55.4%)), fever (67 (39.9%)), vomiting (59 (35.1%)), and headache (27 (16.1%)) were 4 leading complaints in seizure patients.

Neuroimaging done in all seizure patients in the present study suggested abnormal readings in 95 individuals (56.5%), with prevalence of NCC to be 42.9% (72 out of 168 children), whereas a recent study in western Nepal showed 45.9% of seizure patients (111 out of 242 patients) with abnormal brain imaging and prevalence of NCC being 59.5% [16]. Another study done in the same region of Nepal showed that 91% of all NCC cases had seizures as their presenting complaints [21]. This proves that NCC is more common in this part of the country and seizure is an important symptom. Majority (*n* = 56, 77.8%) of NCC children had GTCS when compared to partial seizures but the result was statistically insignificant. The present study did not show any significant difference in the occurrence of GTCS or partial seizures in comparison to normal neuroimaging findings, NCC, or other various abnormal neuroradiological findings.

Neurocysticercosis (72 (42.9%)), idiopathic epilepsy (38 (22.6%)), meningoencephalitis (26 (15.5%)), and febrile convulsions (14 (8.33%)) were four leading disorders in children with seizures in the present study. The findings in a study done by Adhikari et al. from western Nepal were similar to the present study. Still larger studies with larger sample size are required to find the etiological diagnosis in seizure patients more accurately.

Phenytoin was the most commonly preferred antiepileptic drug (58.3%) in treating seizures followed by valproate (32.7%). The reason for widespread use of phenytoin could be because of its easier availability and cheaper cost in developing countries like ours.

## 5. Limitations of the Study

We could not study the outcome of those seizure patients which could have helped to understand the exact disease burden, mortality, and morbidity. We included neither newborns with seizures admitted to NICU nor children having seizures from the outpatient department. This may alter the findings of the present study significantly. Future multicentric studies with larger sample size may be required to solve this problem.

## 6. Conclusion

Children with seizures comprise a significant burden in inpatient department of developing countries with GTCS being more common and having various etiologies. Proper study on clinicodemographic profile of seizures can help in proper understanding of the disease burden and to take appropriate measures for its control.

## Figures and Tables

**Table 1:** Baseline characteristics of children with seizures (GTCS and partial seizures).

Characteristics	Number	GTCS, *n* (%)	Partial, *n* (%)	*P* value
Number of patients, *N* (%)	168	138 (82.1)	30	

Gender				
Male	104	86 (82.7)	18 (17.3)	0.813
Female	64	52 (81.2)	12 (18.8)	

Age (in years)				
0–4	45	37 (82.2)	8 (17.8)	
5–8	44	37 (84.1)	7 (15.9)	0.955
9–12	44	35 (79.5)	9 (20.5)	
13–16	35	29 (82.9)	6 (17.1)	

Mean (±SD)	168	8.2 (4.6)	8.2 (4.1)	

Religion				
Hindu	154	125 (81.2)	29 (18.8)	0.274
Muslim	14	13 (92.9)	1 (7.1)	

**Table 2:** Biochemical parameters of children with GTCS and partial seizures.

Biochemical tests	Number	GTCS (mean ± SD; *n*)	Partial (mean ± SD; *n*)
Haemoglobin (gm/dl)	159	11.5 ± 1.57; 130	11.7 ± 1.07; 29
Total leucocytes count (×10^3^/mm^3^)	159	12.5 ± 6.5; 129	10.4 ± 5.1; 29
Platelets count (lac/mm^3^)	158	3.0 ± 1.4; 119	2.8 ± 0.9; 21
Urea (mg/dl)	140	29.2 ± 22.1; 119	24.0 ± 11.2; 21
Sodium (Na^+^) (mmol/L)	146	136.6 ± 10.1; 122	137.7 ± 2.8; 24
Potassium (K^+^) (mmol/L)	146	4.6 ± 3.7; 122	4.4 ± .6; 24
Random blood sugar (mg/dl)	144	101.1 ± 30.7; 121	105.5 ± 47.5; 23
Calcium (mg/dl)	149	8.7 ± 0.6; 123	8.8 ± 0.5; 26
CSF protein (mg/dl)	52	44.8 ± 19.8; 45	31.2 ± 15.0; 7
CSF lymphocytes (%)	52	97.7 ± 11.3; 45	99.3 ± 1.9; 7
CSF sugar (mg/dl)	52	63.4 ± 15.5; 45	76.4 ± 52.4; 7
CSF polymorphs (%)	46	2.4 ± 11.9; 40	0.8 ± 2.0; 6

**Table 3:** Clinical characteristics of children with generalized tonic-clonic seizures (GTCS) and partial seizures.

Clinical symptoms	Number	GTCS, *n* (%)	Partial, *n* (%)	*P* value
Fever	67	60 (89.5)	7 (10.5)	**0.041**
Vomiting	59	51 (86.4)	8 (13.6)	0.285
Headache	27	24 (88.9)	3 (11.1)	0.318
Meningeal irritation	7	6 (85.7)	1 (14.3)	0.801
Unconsciousness	93	78 (83.9)	15 (16.1)	0.515
Speech disorder	4	2 (50.0)	2 (50.0)	0.089

**Table 4:** Neuroimaging in children with GTCS and partial seizures.

CT scan results	Number	GTCS, *n* (%)	Partial, *n* (%)	*P* value
(a) *Normal*	**73**	**63 (86.3)**	**10 (13.7)**	
(i) Cerebral palsy	3			
(ii) Febrile convulsion	14			
(iii) Idiopathic epilepsy	38			
(iv) Meningoencephalitis	18			
(b) *NCC*	**72**	**56 (77.8)**	**16 (22.2)**	0.402
(c) *Others*	**23**	**18 (78.2)**	**5 (21.7)**	
(i) Meningoencephalitis	8			
(ii) Acute disseminated encephalomyelitis (ADEM)	3			
(iii) Trauma (SDH + SAH)	1			
(iv) Gliotic lesions	2			
(v) ICSOL	2			
(vi) Structural brain abnormalities	7			

**Table 5:** Antiepileptic drugs used in children with GTCS and partial seizures.

Antiepileptic drugs	Number	GTCS, *n* (%)	Partial seizure, *n* (%)
Phenytoin (P)	98	84 (85.7)	14 (14.3)
Sodium valproate (V)	55	41 (74.5)	14 (25.4)
Both P and V combined	11	9 (81.8)	2 (18.2)
More than two drugs	4	4 (100.0)	0 (0.0)

## Abbreviations

GTCS: Generalized tonic-clonic seizures
NCC: Neurocysticercosis
ADEM: Acute disseminated encephalomyelitis
SDH: Subdural hematoma
SAH: Subarachnoid hemorrhage
ICSOL: Intracranial space occupying lesion
CT: Computed tomography.
